# Green tea consumption and incidence of cardiovascular disease in type 2 diabetic patients with overweight/obesity: a community-based cohort study

**DOI:** 10.1186/s13690-024-01242-3

**Published:** 2024-02-02

**Authors:** Bingyue Liu, Shujun Gu, Jin Zhang, Hui Zhou, Jian Su, Sudan Wang, Qian Sun, Zhengyuan Zhou, Jinyi Zhou, Chen Dong

**Affiliations:** 1 Department of Epidemiology and Statistics, School of Public Health, Medical College of Soochow University, Soochow, Jiangsu China; 2 Suzhou Changshu Centers for Disease Control and Prevention, Soochow, China; 3 Suzhou Industrial Park Centers for Disease Control and Prevention, Soochow, China; 4 Jiangsu Provincial Centers for Disease Control and Prevention, Nanjing, China

**Keywords:** Diabetes, Green tea, Cardiovascular diseases, Coronary heart disease, Stroke

## Abstract

**Background:**

Green tea has been reported to be potentially protective against the development of cardiovascular disease (CVD). This study aimed to investigate the association between green tea consumption and incident CVD in type 2 diabetes (T2D) patients with overweight/obesity.

**Methods:**

A total of 4756 Chinese overweight/obese T2D patients were recruited and followed up for 6.27 years. Information on green tea consumption was collected at baseline using interviewer-administered questionnaires. Hazard ratios (HRs) and 95% confidence intervals (CIs) for incident CVD according to green tea consumption were estimated using the Cox proportional hazards model.

**Results:**

Compared with non-habitual consumers, participants who consumed > 5 g/day of green tea leaves reduced the risk of CVD by 29% (95%CI: 0.55–0.92), stroke by 30% (95%CI: 0.51–0.95) and coronary heart disease (CHD) by 40% (95%CI: 0.40–0.89). Similarly, participants who consumed green tea for ≥ 40 years reduced the risk of CVD by 31% (95%CI: 0.54–0.88), stroke by 33% (95%CI: 0.50–0.90) and CHD by 39% (95%CI: 0.42–0.88). Among participants with < 5-year history of T2D, > 5 g/day of tea leaves and > 40 years of tea consumption were associated with 59% (95%CI: 0.23–0.72) and 57% (95%CI: 0.26–0.74) reduced risk of stroke, respectively. However, among participants with ≥ 5-year history of T2D, > 5 g/day of tea leaves and > 40 years of tea consumption were associated with a 50% (95%CI: 0.30–0.82) and 46% (95%CI: 0.35–0.85) reduced risk of CHD, respectively.

**Conclusions:**

Green tea consumption is associated with reduced risk of CVD, stroke, and CHD in overweight/obese T2D patients.

**Supplementary Information:**

The online version contains supplementary material available at 10.1186/s13690-024-01242-3.

**Table:** 

Text box 1. Contributions to the literature	
(1) Green tea consumption is significantly associated with a decreased risk of total cardiovascular disease, coronary heart disease, and stroke in overweight/obese type 2 diabetes patients. (2) Green tea consumption may greatly reduce the risk of stroke in overweight/obese type 2 diabetes patients with < 5-year history of diabetes. (3) Green tea consumption may greatly reduce coronary heart disease risk in overweight/obese type 2 diabetes patients with ≥ 5-year history of diabetes. (4) No significant joint effect of the amount and duration of green tea consumption on the risk of total cardiovascular disease, coronary heart disease, and stroke in overweight/obese type 2 diabetes patients.	

## Introduction

Cardiovascular disease (CVD) is the leading cause of mortality in China. In recent decades, several genetic, lifestyle, dietary and environmental factors have been identified as contributing to the development of CVD, including single nucleotide polymorphisms, obesity, smoking, alcohol consumption, physical activity, heavy metals and others [1]. Diabesity, defined as the combination of diabetes and obesity, is recognized as an important public health problem because of its contribution to cardiac and metabolic dysfunction [2–4]. In a Chinese prospective study included 8006 participants, Kong et al. reported that non-obese diabetic patients had a 42% higher risk of CVD compared with the healthy population. In addition, diabetic patients with obesity had a 78% greater risk of CVD, suggesting that the coexistence of diabetes and obesity may synergistically exacerbate the risk of CVD development [5].

Green tea possesses a great amount of antioxidant components including free amino acids, caffeine and polyphenols. Over the past decade, several population-based studies have reported that green tea consumption is beneficial for several health outcomes, particularly in relation to CVD, including stroke and coronary heart disease (CHD) [6–8]. In a meta-analysis involving 9 studies and 259,267 participants, Pang et al. found that individuals who never consumed green tea had a 19% higher risk of CVD compared with those who consumed one cup per day [9]. Based on two cohort studies of 6517 Chinese adults in Shanghai, Zhao et al. further reported that green tea consumption was inversely associated with the risk of CVD mortality and all-cause mortality [10].

However, the effect of the habitual consumption of green tea on the risk of diabetes and its complications remains uncertain. For instance, based on a cohort study of 0.5 million Chinese adults, Nie et al. reported that daily green tea consumption was significantly associated with a lower risk of type 2 diabetes (T2D), but not associated with the risk of diabetic microvascular complications [11]. In a Mendelian randomization study, Chen et al. reported that green tea consumption did not have a causal effect on T2D and the crucial glycemic profile [12]. However, in another Chinese cohort study, Liu et al. suggested that green tea drinking was associated with an increased risk of T2D, after adjustment for the covariates including age, sex, education, smoking, alcohol intake, physical activity, BMI, and prevalent hypertension [13].

Given that T2D patients with overweight/obesity are at increased risk for CVD, research into effective CVD prevention approaches in these patients is warranted. However, evidence on the association between green tea consumption and CVD risk in overweight/obese T2D patients is still lacking. Therefore, this study is conducted determine the association between green tea consumption and the risk of CVD and its subtypes (CHD and stroke) in overweight/obese T2D patients. The hypothesis of this study is that green tea consumption may protect overweight/obese T2D patients from developing CVD, CHD, and stroke.

## Methods

### Study population

The current work stems from the “Comprehensive Research on the Prevention and Control of the Diabetes” (CRPCD) program, a long-term epidemiological study in Jiangsu (China) that has been ongoing since 2013 and focuses on the risk factors associated with T2D complications [14–17]. Briefly, in the CRPCD program, a total of 10,166 patients with T2D aged 30 years and older were recruited from 30 communities between December 2013 and January 2014. Participants with chronic renal failure, liver cirrhosis, mental illness and severe autoimmune diseases (such as rheumatoid arthritis) were excluded from the CRPCD. At baseline, trained staff conducted face-to-face interviews using an electronic questionnaire and standard physical measurements after participants signed an informed consent. In addition, information about family history of CVD and T2D, comorbidities and medication use for hypertension, T2D and dyslipidemia was collected from each participant. A follow-up examination was carried out between December 2019 and January 2020. The study protocol of CRPCD adhered to the Declaration of Helsinki and was approved by the institutional review board and ethics committee of the Jiangsu Provincial Centers for Disease Control and Prevention (No. 2013026).

To explore the association between green tea consumption and the risk of CVD in overweight/obese T2D patients, 5744 T2D patients aged from 30 to 80 years with a BMI greater than 24 kg/m^2^ at baseline were selected from the CRPCD cohort. Exclusion criteria for the present analysis were as follows: (1) participants with a prior diagnosis of cancer (*n* = 29), coronary heart disease (*n* = 350), or stroke (*n* = 352); (2) those with missing tea consumption information (*n* = 13) and consumed another type of tea (e.g. black tea, oolong tea, dark tea, yellow tea, and other tea) (*n* = 244). Eventually, a total of 4756 participants were included in this study (Supplementary Fig. 1).

### Assessment of green tea consumption

To assess green tea consumption, the following question was asked firstly [10, 11, 13, 14]: Do you like to drink tea (usually ≥ 3 times a week, occasionally < 3 times a week) or not at all? For those who answered “usually ≥ 3 times/week, occasionally < 3 times/week,” the additional questions were asked: (1) When did you start drinking tea (the age of the first drink)? (2) Which kind of tea do you drink most often (green tea, black tea, oolong tea, dark tea, yellow tea, and other tea)? (3) On the days when you drink tea, how many times a day did you usually drink tea in the past year? How often do you change tea leaves during the day? (4) What is the average amount of tea leaves that you add each time (with a picture showing the amount in grams)? For each tea item, serving sizes were multiplied by the consumption frequency to obtain the average daily consumption of tea leaves. Based on these questions, participants were divided into two groups: those who had never drunk tea in the past year, and green tea consumers (those who answered usually ≥ 3 times/week, occasionally < 3 times/week). Next, green tea consumers were further categorized according to the average daily consumption of tea leaves (< 2.5 g/day, 2.5-5 g/day, and > 5 g/day) and the duration of their tea consumption (< 25 years, 25–40 years, and > 40 years), respectively.

### Assessment of covariates

At baseline, information about sociodemographic characteristics (age and sex), lifestyle factors (smoking, alcohol consumption, physical exercises, et al.), personal and family disease history, and current medications was collected from each participant. Anthropometric measures (weight in kg, waist circumference in cm, height in cm, and blood pressure in mmHg) were measured according to standard procedures. Body mass index (BMI) was calculated as follows: BMI = weight [kg]/height squared [m^2^]. T2D was diagnosed according to the American Diabetes Association (ADA) criteria as follows: fasting plasma glucose (FPG) ≥ 7.0 mmol/L (126 mg/dL), or non-fasting glucose level ≥ 11.1 mmol/L (200 mg/dL), or HbA1c ≥ 6.5%, and/or use of antidiabetic medications, with medication use assessed via self-report and medication inventory [18]. In this study, a person who experienced regular exercise below 150 min (3–5 days) per week was considered as having non-regular exercise otherwise it was considered as having regular exercise [19]. Self-reported education attainment was coded as “low education” and “high education”. Low education included no education, primary education, secondary education, and technical or professional school, whereas high education included higher vocational education and university. Annual income was collected as total yearly household income and collapsed into two categories: < 100,000 and ≥ 100,000 CNY. Employment was categorized into currently employed, currently not employed or retired. Marry status was classified as currently married or not currently married. Dietary behavior was assessed by focusing on meat, fruit, and vegetable intake using the questionnaire [14–17]. Family CVD history was defined as self-reported coronary artery disease, heart failure, stroke, or peripheral vascular disease in parents and other family members. Family history of T2D was defined as the presence of T2D in at least one first- or second-degree relative. Central obesity was defined as waist circumference ≥ 90 cm in males and ≥ 85 cm in females according to the Chinese adult weight criteria (WS/T 428–2013) [20]. The presence of one or more complications related to diabetes, such as retinopathy, neuropathy, nephropathy, and diabetic foot ulcers, was considered to indicate complications from T2D [21, 22].

At baseline, a fasting blood sample was collected from each participant and a serum or plasma sample was obtained via centrifugation at 2000× g for 10 min at 4 °C. Serum total cholesterol (TC), triglycerides (TG), and high-density lipoprotein-cholesterol (HDL-C), were measured using biochemical reagent kits on an automated biochemical analyzer (Hitachi, Tokyo, Japan) according to the manufacturer’s introduction [23]. The coefficient of variation was less than 10% for all methods, both intra- and inter-assay. The low-density lipoprotein-cholesterol (LDL-C) level was calculated using the Friedewald equation (TC minus HDL-C minus TG/5) [24]. FPG was measured using the hexokinase method on a Roche 702 instrument with commercial reagents. The coefficients of variation were less than 10% for both intra- and inter-assay measurements [25]. Glycosylated hemoglobin (HbA1c) was measured with the BIO-RAD VARIANT II. The intra-assay and inter-assay coefficients of variation were less than 7.9 and 9.9%, respectively [26].

### Assessment of study outcomes

All participants were followed up after the baseline survey until 31/12/2020. CVD outcomes were collected using a structured questionnaire by the trained physician and the International Classification of Diseases codes (ICD-9 and ICD-10), which included fatal and non-fatal coronary heart disease (CHD) events [myocardial infarction (I21, I22)], and fatal and non-fatal stroke events [subarachnoid hemorrhage (I60), hemorrhagic stroke (I61), cerebral ischemic stroke (I63), not specified as hemorrhage or infarction (I64)] [27]. For fatal events, the date and cause of death were obtained from the Cause of Death Statistics from the Changshu Industrial Park Centers for Disease Control and Prevention.

### Statistical analysis

Data are presented as mean values ± standard deviation (SD) or percentages. Continuous data were compared using the Student t-test, Mann–Whitney U test or Kruskal-Wallis H test, as appropriate. Categorical variables were compared using the χ^2^ Chi-square test. *P* values were adjusted using Benjamini–Hochberg (BH) method [28]. In this study, the missing baseline measurements including serum lipid profiles (*n* = 24), vegetable consumption (*n* = 73), fruit consumption (*n* = 70), and meat consumption (*n* = 64)) were imputed using the multiple interpolation method [29]. For each participant, person-years of follow-up were calculated from the date of the return of the baseline questionnaire until the date of the first event related to CVD (CHD and stroke), or until the date of death from any cause, loss to follow-up, or December 31, 2020, whichever occurred first. We considered participants without information regarding the cause of mortality who were lost to follow-up as alive after loss.

Multivariable Cox proportional hazard models were used to estimate hazard ratios (HRs) and 95% confidence intervals (CIs) of total CVD risk, comparing the green tea consumers to the participants without habitual green tea consumption. The proportional hazards assumption was assessed using Schoenfeld residuals. Next, HRs (95% CIs) was calculated for total CVD, CHD, and stroke probability according to the categories of the daily amount of tea leaves intake (< 2.5, 2.5-5, and > 5 g/day) and the duration of green tea consumption (< 25, 25–40, and > 40 years). In this study, the potential confounders were determined by a directed acyclic graph (DAG) (Supplementary Fig. 2) [30, 31]. Model 1 was the crude model. Model 2 was adjusted for age (continuous), sex (male and female), smoking status (no or yes), alcohol consumption status (no or yes), BMI (continuous), annual income (< 100,000 or ≥ 100,000 CNY), education (lower or higher education), employment (employed or not employed), marry status (currently married or not currently married), physical exercise (regular or non-regular physical exercise), SBP (continuous), DBP (continuous), dyslipidemia (yes or no), hypertension (yes or no), lipid-lowering drugs (yes or no), antihypertensive drugs (yes or no), oral hypoglycaemic agents (yes or no), family history of CVD (yes or no), family history of T2D (yes or no), times of weekly meat/fruit/vegetable consumption (< 4 or ≥ 4times, average 100 g per time), and all listed risk factors for Model 3 [HbA1c (continuous), FPG (continuous), T2D complications (yes or no), and T2D duration (continuous)]. The Wald test was used for linear trends evaluation by assigning the median intake within each group and adding them as continuous variables in the models. In this study, the use of insulin, supplementing the model 3 with TC and TG, or substituting BMI as the adjustment variable with waist circumference was further assessed through a sensitivity analysis. Moreover, the sensitivity analysis was used to examine the associations between green tea intake and total CVD, stroke, and CHD in T2D patients with central obesity. In addition, the E-value method was employed to conduct a sensitivity analysis of potential unmeasured confounders in this study. The E-value was defined as the minimum strength of association on the risk ratio scale that an unmeasured confounder must have with both the exposure and the outcome to fully explain an observed association, conditional on the measured covariates. the point estimate and the lower limit of the 95% CI were computed for the E-value as previously described [32, 33].

To detect effect modification, subgroup analyses were conducted according to baseline characteristics including sex, age, annual income by household, smoking status, and T2D duration. Possible interactions between green tea consumption and risk factors, concerning the incidence of total CVD, CHD, and stroke, were tested by introducing interaction terms in the multivariate model (one at a time).

All analyses were performed using R software version 4.1.0. Two-tailed *P* < 0.05 was considered statistically significant in the current study.

## Results

During the 29,818.62 person-years of follow-up, a total of 915 new CVD cases (625 stroke cases and 398 CHD cases) were documented, with a crude incidence rate of 30.69 cases/1000 person-years. Compared with participants who did not consume green tea, green tea consumers were more likely to be male, younger, smokers, alcohol drinkers, have lower waist circumference, have regular physical activities, have a longer duration of T2D, have higher blood pressure, have higher levels of FPG, HbA1c and TC, and have lower HDL-C levels. They also appeared to be more physically active and consumed more red meat and fresh fruit, but less vegetables. However, there were no significant differences in the family history of CVD and T2D between the participants with and without habitual green tea consumption (Table 1).

**Table 1:** Baseline characteristics of T2D patients with overweight/obesity

	Non- consumption (*n* = 2933)	Green tea consumption (*n* = 1823)	P_1_-values/ Adjusted P_1_-values	Daily green tea leaves consumption (g/day)			*P*_2_-values/ Adjusted *P*_2_-values	Duration of green tea consumption (years)			*P*_3_-values/ Adjusted *P*_3_-values
				< 2.5	2.5–5	> 5		< 25	25–40	> 40	
Age, years	63.52 ± 8.47	62.11 ± 9.08	< 0.001/0.002	62.13 ± 9.24	62.10 ± 8.85	61.73 ± 8.98	< 0.001/0.002	56.84 ± 10.34	60.10 ± 6.89	69.62 ± 4.96	< 0.001/0.002
Male, n (%)	443 (15.1)	1447 (79.4)	< 0.001/0.002	449 (64.3)	634 (84.6)	558 (90.0)	< 0.001/0.002	304 (57.3)	809 (86.0)	528 (88.7)	< 0.001/0.002
BMI, kg/m^2^	26.90 ± 2.32	26.85 ± 2.29	0.472/0.505	26.88 ± 2.37	26.82 ± 2.20	26.89 ± 2.30	0.853/0.853	26.94 ± 2.40	26.82 ± 2.22	26.85 ± 2.29	0.762/0.762
waist circumference, cm	88.55 ± 7.83	91.03 ± 7.48	< 0.001/0.002	90.29 ± 7.65	90.93 ± 7.51	92.02 ± 7.13	< 0.001/0.002	90.46 ± 7.60	90.97 ± 7.51	91.64 ± 7.28	< 0.001/0.002
Systolic blood pressure, mmHg	151.75 ± 19.08	150.46 ± 18.75	0.022/0.036	151.48 ± 18.95	149.31 ± 18.23	150.20 ± 18.32	0.008/0.017	148.50 ± 19.09	150.02 ± 18.45	152.38 ± 17.92	< 0.001/0.002
Diastolic blood pressure, mmHg	81.21 ± 9.57	84.51 ± 10.16	< 0.001/0.002	83.96 ± 10.31	84.44 ± 9.95	85.28 ± 10.11	< 0.001/0.002	84.93 ± 10.77	85.64 ± 9.90	82.42 ± 9.57	< 0.001/0.002
smoker, n (%)	169 (5.8)	827 (45.4)	< 0.001/0.002	242 (34.7)	367 (49.0)	328 (52.9)	< 0.001/0.002	183 (34.5)	494 (52.5)	260 (43.7)	< 0.001/0.002
alcohol drinker, n (%)	210 (7.2)	836 (45.9)	< 0.001/0.002	269 (38.5)	349 (46.6)	338 (54.5)	< 0.001/0.002	178 (33.5)	489 (52.0)	289 (48.6)	< 0.001/0.002
Low education, n (%)	2918 (99.5)	1757 (96.4)	< 0.001/0.002	614 (97.5)	627 (96.0)	516 (95.6)	< 0.001/0.002	455 (96.8)	788 (96.5)	514 (95.9)	< 0.001/0.002
Low-income households, n (%)	2294 (78.2)	1339 (73.5)	< 0.001/0.002	495 (78.6)	477 (73.0)	367 (68.0)	< 0.001/0.002	344 (73.2)	585 (71.6)	410 (76.5)	< 0.001/0.002
Currently employed, n (%)	1133 (38.6)	881 (48.3)	< 0.001/0.002	300 (47.6)	321 (49.2)	260 (48.1)	< 0.001/0.002	273 (58.1)	476 (58.3)	132 (24.6)	< 0.001/0.002
Currently married, n (%)	2910 (99.2)	1800 (98.7)	0.138/0.178	626 (99.4)	640 (98.0)	534 (98.9)	0.029/0.046	464 (98.7)	809 (99.0)	527 (98.3)	0.228/0.263
Regular physical activities, n (%)	1749 (59.6)	1131 (62.0)	0.105/0.148	372 (59.0)	425 (65.1)	334 (61.9)	0.054/0.077	260 (55.3)	508 (62.2)	363 (67.7)	< 0.001/0.002
Red meat consumption ≥ 400 g/week, n (%)	816 (27.8)	677 (37.1)	< 0.001/0.002	227 (36.0)	242 (37.1)	208 (38.5)	< 0.001/0.002	177 (37.7)	296 (36.2)	204 (38.1)	< 0.001/0.002
Fresh fruits consumption ≥ 400 g/week, n (%)	507 (17.3)	368 (20.2)	0.013/0.024	117 (18.6)	130 (19.9)	121 (22.4)	0.027/0.045	104 (22.1)	164 (20.1)	100 (18.7)	0.040/0.048
Fresh vegetables consumption ≥ 400 g/week, n (%)	2769 (94.4)	1683 (92.3)	0.005/0.010	589 (93.5)	602 (92.2)	492 (91.1)	0.012/0.021	429 (91.3)	753 (92.2)	501 (93.5)	0.016/0.021
Hypertension, n (%)	2524 (86.1)	1567 (86.0)	0.959/0.968	607 (87.0)	642 (85.7)	526 (84.8)	0.732/0.757	419 (78.9)	809 (86.0)	547 (91.9)	< 0.001/0.002
Antihypertensive drugs, n (%)	1934 (65.9)	1169 (64.1)	0.213/0.264	447 (64.0)	463 (61.8)	413 (66.6)	0.143/0.172	304 (57.3)	590 (62.7)	429 (72.1)	< 0.001/0.002
Dyslipidemia, n (%)	1430 (48.8)	956 (52.4)	0.015/0.026	382 (54.7)	371 (49.5)	333 (53.7)	0.010/0.020	304 (57.3)	512 (54.4)	270 (45.4)	< 0.001/0.002
Lipid-lowering drugs, n (%)	53 (1.8)	57 (3.1)	0.004/0.009	18 ( 2.9)	13 ( 2.0)	26 ( 4.8)	< 0.001/0.002	11 (2.3)	29 (3.5)	17 (3.2)	0.014/0.019
Oral hypoglycaemic agents, n (%)	2345 (80.0)	1491 (81.8)	0.128/0.173	512 (81.3)	532 (81.5)	447 (82.8)	0.404/0.466	378 (80.4)	676 (82.7)	437 (81.5)	0.322/0.358
Family history of CVD, n (%)	178 (6.1)	127 (7.0)	0.243/0.290	60 (8.6)	44 (5.9)	44 (7.1)	0.080/0.104	37 (7.0)	67 (7.1)	44 (7.4)	0.483/0.518
Family history of T2D, n (%)	776 (26.5)	503 (27.6)	0.410/0.454	165 (26.2)	188 (28.8)	150 (27.8)	0.604/0.671	165 (35.1)	219 (26.8)	119 (22.2)	< 0.001/0.002
T2D duration, years	6.26 ± 5.23	6.59 ± 5.52	0.041/0.064	6.50 ± 5.52	6.63 ± 5.58	6.79 ± 5.39	0.074/0.101	5.91 ± 5.13	6.49 ± 5.29	7.51 ± 6.01	< 0.001/0.002
T2D complications, n (%)	445 (15.2)	275 (15.1)	0.968/0.968	76 (12.1)	103 (15.8)	96 (17.8)	0.051/0.077	57 (12.1)	116 (14.2)	102 (19.0)	0.017/0.021
HbA1c, %	7.49 ± 1.46	7.73 ± 1.53	< 0.001/0.002	7.71 ± 1.58	7.71 ± 1.48	7.90 ± 1.63	< 0.001/0.002	7.71 ± 1.60	7.87 ± 1.57	7.65 ± 1.51	< 0.001/0.002
FPG, mmol/L	8.59 ± 2.45	9.01 ± 2.66	< 0.001/0.002	9.01 ± 2.73	9.00 ± 2.70	9.03 ± 2.51	< 0.001/0.002	9.22 ± 2.71	9.16 ± 2.76	8.61 ± 2.40	< 0.001/0.002
TG, mmol/L	2.13 ± 1.69	2.22 ± 1.96	0.095/0.140	2.27 ± 2.05	2.15 ± 1.98	2.29 ± 1.99	0.114/0.143	2.39 ± 2.09	2.30 ± 2.04	1.98 ± 1.86	< 0.001/0.002
TC, mmol/L	5.32 ± 1.15	5.24 ± 1.06	0.009/0.017	5.30 ± 1.07	5.18 ± 0.98	5.24 ± 1.14	0.011/0.021	5.33 ± 1.06	5.22 ± 1.03	5.18 ± 1.11	0.005/0.007
LDL - C, mmol/L	3.18 ± 0.89	3.16 ± 0.88	0.289/0.320	3.18 ± 0.92	3.14 ± 0.85	3.16 ± 0.88	0.674/0.722	3.19 ± 0.83	3.16 ± 0.90	3.14 ± 0.90	0.625/0.647
HDL - C, mmol/L	1.46 ± 0.35	1.36 ± 0.34	< 0.001/0.002	1.37 ± 0.34	1.37 ± 0.34	1.34 ± 0.33	< 0.001/0.002	1.35 ± 0.33	1.34 ± 0.33	1.41 ± 0.35	< 0.001/0.002

*P*_*1*_: Continuous data were compared using the Student t-test, and Mann–Whitney U test, as appropriate. Categorical variables were compared using the χ^2^ Chi-square test

*P*_*2*_&*P*_*3*_: Continuous data were compared using the Kruskal-Wallis H test. Categorical variables were compared using the χ^2^ Chi-square test

CVD: Cardiovascular disease; T2D: Type 2 diabetes; FPG: Fasting plasma glucose; TC: Total cholesterol; TG: Triglycerides; LDL-C: low-density lipoprotein-cholesterol; HDL-C: high-density lipoprotein-cholesterol.

As shown in Table 2 and Supplementary Table 1, after adjustment for potential confounders, green tea consumption was significantly associated with a reduced risk of CVD in overweight/obese T2D patients (HR: 0.76, 95%CI: 0.63–0.91). Consistently, green tea consumption was associated with a significant reduction in the subsequent risk of stroke (adjusted HR: 0.77, 95% CIs: 0.62–0.96) and CHD (adjusted HR: 0.68, 95% CIs: 0.52–0.90).

**Table 2:** Association between green tea consumption and the risk of CVD, stroke, and CHD

	Total CVD		Stroke		CHD	
	HR (95% CI)	Adjusted *P*_values_	HR (95% CI)	Adjusted *P*_values_	HR (95% CI)	Adjusted *P*_values_
Green tea consumption						
Non- consumption	1		1		1	
Green tea consumers	0.76 (0.63–0.91)	0.003	0.77 (0.62–0.96)	0.020	0.68 (0.52–0.90)	0.006
Daily tea leaves consumption						
Non- consumption	1		1		1	
< 2.5 g/day	0.79 (0.63–0.98)	0.034	0.81 (0.62–1.06)	0.127	0.72 (0.51–1.01)	0.06
2.5–5 g/day	0.76 (0.60–0.96)	0.024	0.78 (0.58–1.04)	0.094	0.70 (0.49–1.01)	0.054
> 5 g/day	0.71 (0.55–0.92)	0.008	0.70 (0.51–0.95)	0.022	0.60 (0.40–0.89)	0.011
*P* trend		0.019		0.036		0.020
Duration of consumption						
Non- consumption	1		1		1	
< 25 years	0.77 (0.58–1.01)	0.056	0.77 (0.55–1.07)	0.118	0.74 (0.49–1.12)	0.152
25–40 years	0.82 (0.65–1.03)	0.089	0.88 (0.67–1.16)	0.359	0.71 (0.49–1.01)	0.054
> 40 years	0.69 (0.54–0.88)	0.002	0.67 (0.50–0.90)	0.008	0.61 (0.42–0.88)	0.009
*P* trend		0.003		0.015		0.005

Models were adjusted for covariates in age, sex, smoking status, alcohol consumption status, BMI, annual income, education, employment, marital status, physical exercise, SBP, DBP, dyslipidemia, hypertension,lipid-lowering drugs, antihypertensive drugs, oral hypoglycaemic agents, family history of CVD, family history of T2DM, times of weekly meat/fruit/vegetable consumption, HbA1c, FPG, diabetes duration, and diabetes complications.

The average amount of daily green tea leaves consumption was then processed as a categorical variable (< 2.5, 2.5-5, and > 5 g/day). Compared with non-habitual green tea consumers, the risk of total CVD, stroke, and CHD was significantly reduced in participants who consumed > 5 g/day of green tea leaves, with adjusted HRs (95% CIs) of 0.71 (0.55–0.92) for total CVD, 0.70 (0.51–0.95) for stroke, and 0.60 (0.40–0.89) for CHD (Table 2 and Supplementary Table 1). The sensitivity analysis showed that daily average tea intake remaining inversely associated with total CVD, stroke, and CHD risk, even in individuals with centrally obese participants, or further adjustment for the use of insulin, TC and TG, the substitution of waist circumference for the BMI (Supplementary Tables 2–4). In addition, most of the E-values for the risk of total CVD and the daily tea leaves consumption were higher than the HR values for the traditionally important CVD risk factors such as hypertension, smoking, alcohol consumption, and others. This suggested that the association between daily consumption of green tea leaves and the risk of CVD was not significantly confounded by unmeasured confounders (Supplementary Tables 5–6).

Furthermore, after full adjustment for potential confounders, overweight/obese T2D patients who maintained their green tea consumption habits for more than 40 years had a considerably lower risk of total CVD (HR: 0.69, 95%CI: 0.54–0.88), stroke (HR: 0.67, 95%CI: 0.50–0.90), CHD (HR: 0.61, 95%CI: 0.42–0.88), respectively (Table 2 and Supplementary Table 1). Even in the participants with central obesity, or further adjustment for the use of insulin, TC and TG, or for the substitution of waist circumference for BMI (Supplementary Tables 7–9). In addition, the E-values for CVD risk and duration time of green tea consumption were higher than the HR values for the traditional CVD risk factors, suggesting that the association between the duration time of green tea consumption and the risk of CVD was relatively stable (Supplementary Tables 5–6).

**Fig. 1:**
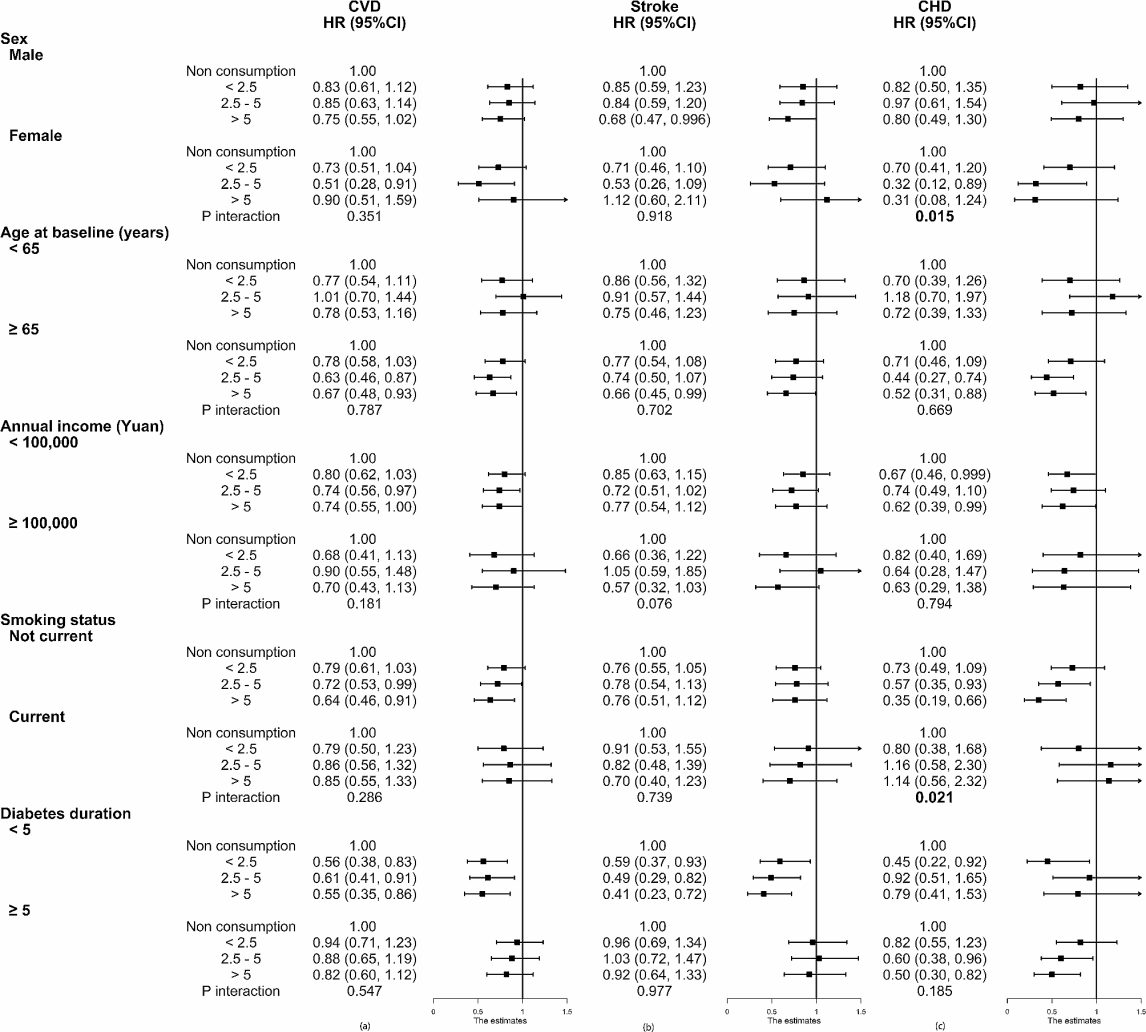
Subgroup analysis of associations between daily consumption of green tea leaves and the risk of total CVD (**a**), stroke (**b**), and CHD (**c**) according to potential baseline risk factors. Values were obtained from Cox proportional hazards analysis. Except for the baseline stratifying variable, the model was adjusted for the same covariates as in the model of Table 2

The results of the subgroup analysis revealed that a stronger inverse association between the average amount of daily tea leaves consumption and incident CVD was observed in elders (≥ 65 years) (HR: 0.67, 95%CI: 0.48–0.93), non-smokers (HR: 0.64, 95%CI: 0.46–0.91) and participants with < 5-year history of T2D (HR: 0.55, 95%CI: 0.35–0.86) (Fig. 1a). As shown in Fig. 1b, associations for daily tea leaves consumption with incident stroke tended to be significant in males (HR: 0.68, 95%CI: 0.47–0.996), elders (≥ 65 years) (HR: 0.66, 95%CI: 0.45–0.99), and participants with < 5-year history of T2D (HR: 0.41, 95%CI: 0.23–0.72). For the influence of daily tea leaves consumption on the risk of CHD, a significant association was observed in elders (≥ 65 years) (HR: 0.52, 95%CI: 0.31–0.88), with lower annual income (HR: 0.62, 95%CI: 0.39–0.99), non-smokers (HR: 0.35, 95%CI: 0.19–0.66), and participants with ≥ 5-year history of T2D (HR: 0.50, 95%CI: 0.30–0.82) (Fig. 1c). In addition, in the multivariate model, there is a significant interaction between sex and daily tea leaves consumption on the future CHD probability (*P-*_*interaction*_ = 0.015), as well as smoking status (*P-*_*interaction*_ = 0.021).

**Fig. 2:**
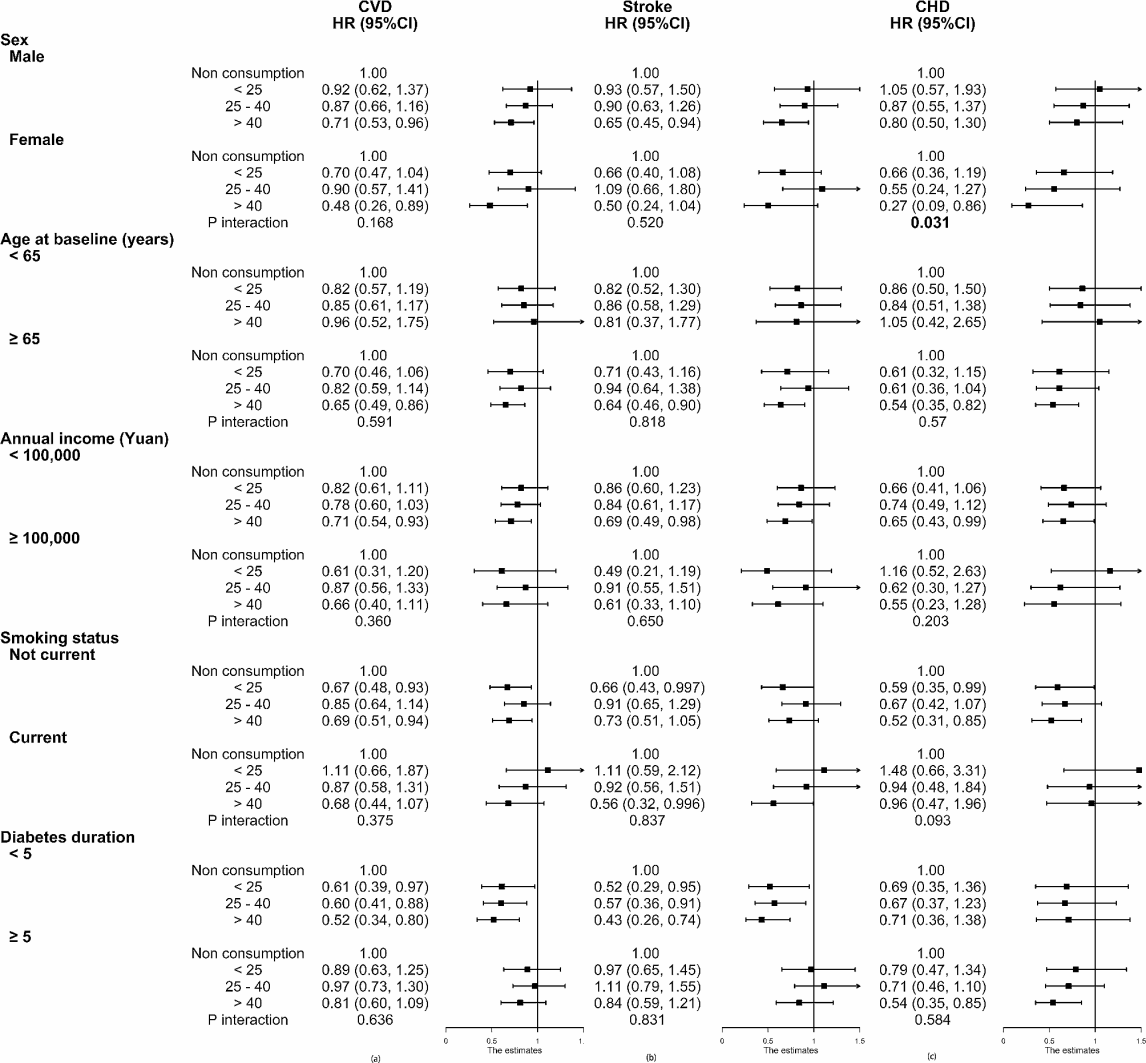
Subgroup analyses of associations between duration of green tea consumption and the risk of CVD (**a**), stroke (**b**), and CHD (**c**) according to potential baseline risk factors. Values were obtained from Cox proportional hazards analysis. Except for the baseline stratifying variable, the model was adjusted for the same covariates as in the model of Table 2

As shown in Fig. 2a, the inverse association between the duration of green tea consumption and total CVD incidence were observed in elders (≥ 65 years) (HR: 0.65, 95%CI: 0.49–0.86), with lower annual income for household (HR: 0.71, 95%CI: 0.54–0.93), non-smokers (HR: 0.69, 95%CI: 0.51–0.94), and the participants with < 5-year history of T2D (HRs: 0.52, 95%CI: 0.34–0.80). In addition, a statistical association between the duration of green tea consumption and incident stroke was observed in males (HR: 0.65, 95%CI: 0.45–0.94), elders (≥ 65 years) (HR: 0.64, 95%CI: 0.46–0.90), with lower annual income (HR: 0.69, 95%CI: 0.49–0.98), smokers (HR: 0.56, 95%CI: 0.32–0.996), and participants with < 5-year history of T2D (HR: 0.43, 95%CI: 0.26–0.74) (Fig. 2b). As shown in Fig. 2c, associations for duration of green tea consumption with incident CHD tended to be more strongly inverse in females (HR: 0.27, 95%CI: 0.09–0.86), elders (≥ 65 years) (HR: 0.54, 95%CI: 0.35–0.82), with lower annual income (HR: 0.65, 95%CI: 0.43–0.99), non-smokers (HR: 0.52, 95%CI: 0.31–0.85) and participants with ≥ 5-year history of T2D (HR: 0.54, 95%CI: 0.35–0.85). In the multivariate model, there is a significant interaction between sex and the duration of green tea consumption on the future CHD probability (*P-*_*interaction*_= 0.031).

As shown in Table 3, there was no significant joint effect of the amount and duration of green tea consumption on the risk of total CVD, stroke, and CHD in overweight/obese T2D patients during follow-up, as compared to the participants who have less than 2.5 g/day of green tea leaves and less than 30 years of green tea consumption.

**Table 3:** The combined effect of the amount and duration of green tea consumption on the risk of CVD, stroke, and CHD

Daily consumption of tea leaves	Duration of tea consumption	Cases	Cases/ PYs	HR (95% CI)	Adjusted *P*_− values_
			(/1000)		
Total CVD					
< 2.5 g/day	< 30 years	40	21.98	1	
	≥ 30 years	76	35.53	1.08 (0.73–1.60)	0.699
≥ 2.5 g/day	< 30 years	52	21.16	0.96 (0.63–1.46)	0.847
	≥ 30 years	168	33.25	0.99 (0.691.43)	0.977
Stroke					
< 2.5 g/day	< 30 years	28	15.39	1	
	≥ 30 years	53	24.78	1.08 (0.67–1.72)	0.761
≥ 2.5 g/day	< 30 years	36	14.65	0.98 (0.59–1.62)	0.937
	≥ 30 years	112	22.16	0.95 (0.61–1.46)	0.805
CHD					
< 2.5 g/day	< 30 years	18	9.89	1	
	≥ 30 years	30	14.02	0.95 (0.52–1.73)	0.862
≥ 2.5 g/day	< 30 years	20	8.14	0.80 (0.42–1.53)	0.507
	≥ 30 years	69	13.65	0.90 (0.52–1.54)	0.700

Models were adjusted for covariates in age, sex, smoking status, alcohol consumption status, BMI, annual income, education, employment, marital status, physical exercise, SBP, DBP, dyslipidemia, hypertension,lipid-lowering drugs, antihypertensive drugs, oral hypoglycaemic agents, family history of CVD, family history of T2DM, times of weekly meat/fruit/vegetable consumption, HbA1c, FPG, diabetes duration, and diabetes complications

## Discussion

In this large community-based cohort study, the results reported novel evidence on the protective effects of green tea consumption on the risks of total CVD, CHD, and stroke in overweight/obese T2D patients. The following key findings were identified: (1) green tea consumption was significantly associated with a decreased risk of total CVD, CHD, and stroke in overweight/obese T2D patients. Furthermore, drinking more and drinking for longer resulted in a lower risk of total CVD, CHD, and stroke; (2) in the patients with < 5-year history of T2D, green tea consumption greatly decreased the risk of stroke; (3) in the patients with ≥ 5-year history of T2D, green tea consumption significantly reduced future CHD probability; (4) there was no significant joint effect of the amount and duration of green tea consumed on the risk of total CVD, CHD, and stroke in overweight/obese T2D patients. As the most popular consumed beverages in China, green tea has high concentrations of tea polyphenols, theaflavin and other antioxidants with posited beneficial properties. For example, Li et al. have reported that theaflavin attenuates cerebral ischemia/reperfusion injury by abolishing miRNA‑128‑3p‑mediated Nrf2 inhibition and reducing oxidative stress [34]. Therefore, our present results support the notion that habitual consumption of green tea may have protective effects against the development of CVD, CHD, and stroke.

Over the past decades, several epidemiology studies have been conducted to explore the association between habitual tea consumption and the risks of CVD, stroke, and CHD. Although some studies supported that consumption of green tea was associated with a reduce risk of myocardial infarction and stroke in diabetics [6, 35–38], the results from the China Kadoorie Biobank study reported that diabetic patients who consumed green tea were not associated with the risk of macrovascular complications [11]. In the present study, the results showed that green tea consumption was associated with a lower risk of CVD, CHD, and stroke. Given that the risks of CVD, CHD, and stroke were significantly increased in overweight/obese T2D patients compared with the non-obese diabetic patients and general population [5, 39, 40], we speculate that the differences reported in different studies may be due to differences in the characteristics of the study populations. In addition, although we carefully adjusted for the covariates, residual confounding (e.g., the production region and manufacturing technique of tea leaves) may also contribute to the inconsistent associations between green tea consumption and the subsequent risk of CVD, CHD, and stroke [41].

In this study, the results showed that green tea consumption was significantly associated with a reduced risk of stroke but not with incident CHD in patients with < 5-year history of T2D. However, in the participants with ≥ 5-year history of T2D, green tea consumption could protect against the development of CHD but did not significantly affect the risk of stroke. Several studies have reported different incidence rates of stroke and CHD in diabetic patients [42–45]. For example, the Emerging Risk Factors Collaboration meta-analysis of 102 prospective studies with 8.5 million person-years of follow-up showed that T2D increased the risks of ischemic and hemorrhagic stroke by 2.27 and 1.56 times, respectively [46]. In addition, because the risk of stroke and CHD may differ among the diabetic patients during the disease progression [47, 48], future studies should be conducted to validate the results of our present study.

The results from the stratified analysis showed that the inverse associations of green tea consumption with CVD were strengthened among non-smokers, older adults (≥ 65 years), and participants with < 5-year history of T2D. Furthermore, our study revealed for the first time the potential modifying effects of sex and smoking on the association between green tea consumption and CHD. A possible explanation for these findings is that habitual green tea consumers with CVD generally tend to have worse lifestyle habits such as cigarette smoking and alcohol consumption [38, 49–51]. In a Chinese cohort study involving 164,681 male participants, Liu et al. reported that habitual green tea consumption was inversely associated with CVD in non-smokers and non-regular alcoholic consumers [49], which was consistent with our findings.

To the best of our knowledge, this study is the first prospective cohort study to investigate the effect of green tea consumption on the risk of total CVD, CHD, and stroke in overweight/obese T2D patients. The strengths of our study included a prospective design, a large sample size, long-term follow-up, and information on various covariates. In addition, we measured the average daily amount (g/day) and duration of green tea consumption, which might better reflect the intake of active biochemical from tea. However, some limitations should be mentioned. First, this study used self-reported green tea consumption, which might have misrepresented true consumption due to recall bias. In addition, green tea consumption and other covariates were measured only at the baseline. The levels may have changed over time before the CVD events. Second, our cohort of middle-aged and older Chinese overweight/obese T2D patients, might limit the generalizability of our findings to other populations with different age structures and various comorbidities. Third, although our results suggested that green tea consumption might be protective against CVD, CHD, and stroke in overweight/obese T2D patients, the effects of other types of tea (e.g., black tea) on CVD, CDH, and stroke risk have not been carefully assessed due to the limited sample sizes (Supplementary Table 10). Fourth, despite the availability of numerous confounders that have been corrected, we cannot exclude the presence of residual confounders, such as the location of the participants (urban/rural), dietary habits (sodium, sugar and others), environmental factors (air pollution, heavy metal exposure and others), the daily green tea intake time, the type of green tea and others. However, the E-value was calculated to test for the potential interference caused by unmeasured confounders, and the results indicated that the association between green tea consumption and the risk of CVD in overweight/obese T2D patients with was reliably stable. Finally, the mechanisms underlying our current findings are not fully understood. For example, we observed that the duration of green tea consumption was inversely associated with CVD in non-alcoholic drinkers. However, the average daily consumption of green tea leaves was significantly associated with a reduced risk of CVD among current alcoholic consumers. In addition, we observed an inverse association between green tea consumption and CVD in the participants with hypertension, but not in those with normotension, suggesting a complex synergistic effect between traditional CVD risk factors and green tea consumption that warrants further exploration.

## Conclusions

In summary, this community-based cohort study revealed that green tea consumption has protective effects on the development of total CVD, CHD, and stroke in overweight/obese T2D patients. If our present findings are validated in other populations, it will support the recommendation of green tea consumption as a healthy habit to protect against CVD in T2D patients with overweight/obesity.

### Electronic supplementary material

Below is the link to the electronic supplementary material.

Supplementary Material 1

## Data Availability

All data and materials presented in this research paper are available by contacting the corresponding author upon request.

## Abbreviations

CVD: Cardiovascular disease
CHD: Coronary heart disease
T2D: Type 2 diabetes
HR: Hazard ratios
95% CIs: 95% confidence intervals
BMI: Body mass index
SBP: Systolic blood pressure
DBP: Diastolic blood pressure
FPG: Fasting plasma glucose
WC: Waist circumference
TC: Total cholesterol
TG: Triglycerides
LDL-C: Low-density lipoprotein-cholesterol
HDL-C: High-density lipoprotein-cholesterol
BH: Benjamini–Hochberg
DAG: Directed acyclic graph
