# Supplementary material for: Green tea consumption and incidence of cardiovascular disease in type 2 diabetic patients with overweight/obesity: a community-based cohort study
Source: Arch Public Health. 2024 Feb 2;82:18. doi: 10.1186/s13690-024-01242-3 (PMC10835928; doi:10.1186/s13690-024-01242-3)
Supplement: Supplementary file 1 — Supplementary Material 1 [file 13690_2024_1242_MOESM1_ESM.docx]

**
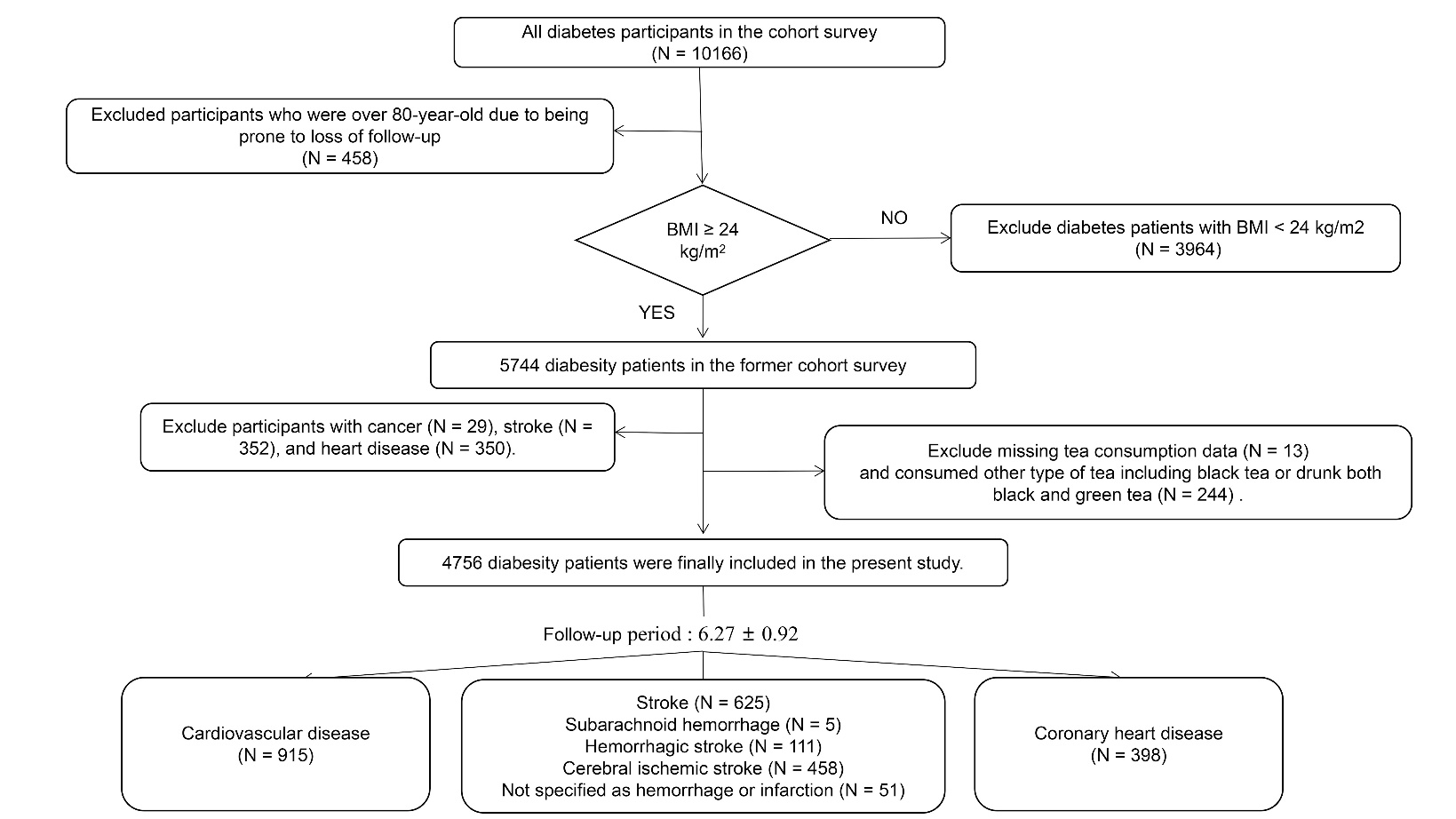
**

**Supplementary Figure 1**: Flow chart of the study, incorporated into the study were patients with CVD (915), consisting of 517 patients with stroke, 290 patients with CHD, and 108 patients with both stroke and CHD.

**
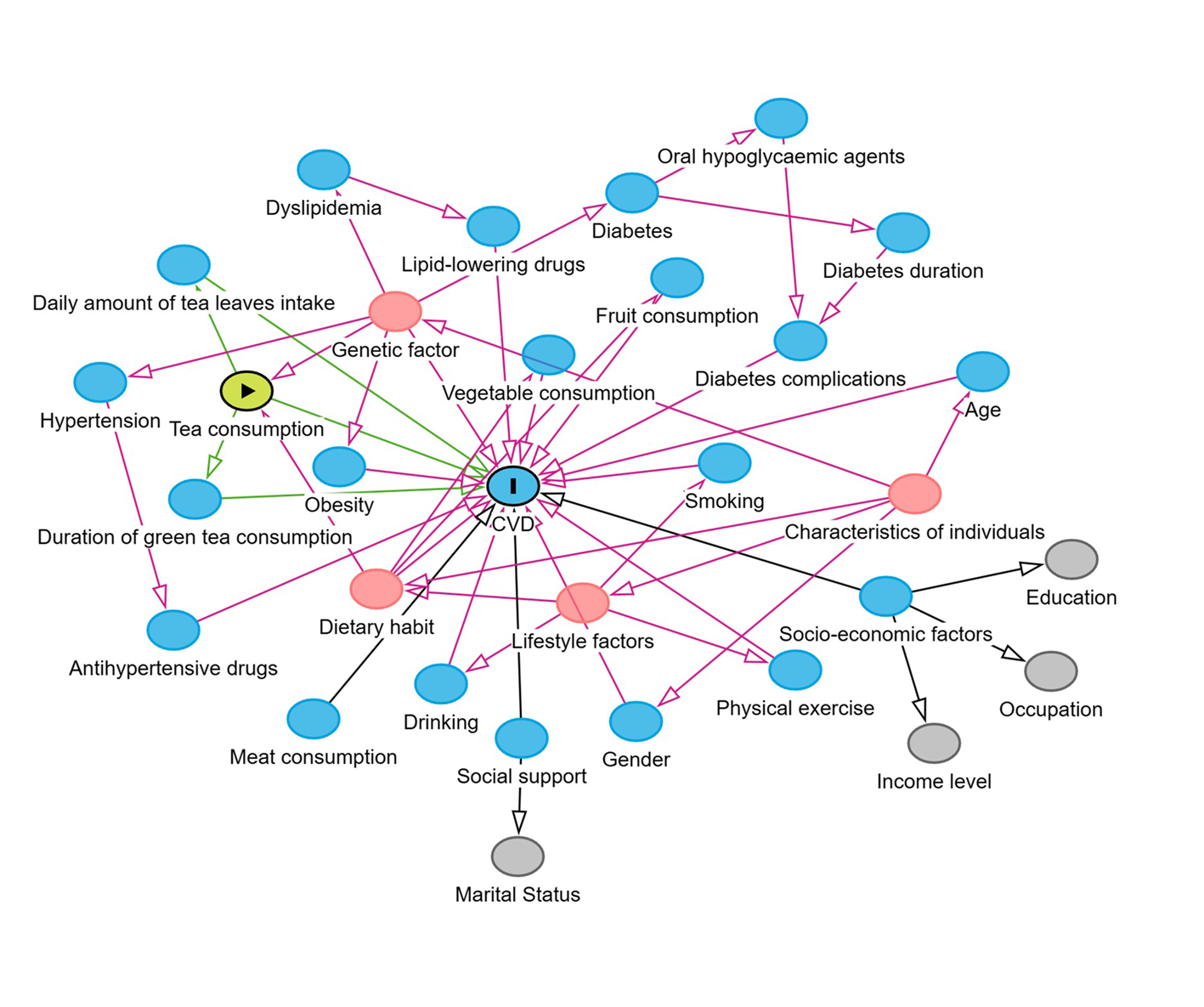
**

**Supplementary Figure 2**: Directed Acyclic Graph A directed acyclic graph represents associations between covariates and primary exposure and outcome. Pink circles represent ancestors of the exposure and outcome (ie, confounders), blue circles represent ancestors of the outcome (ie, causal determinants of the outcome).

**Supplementary Table 1:** Association between green tea consumption and the risk of CVD, stroke, and CHD

|  | **Model 1** | | **Model 2** | | **Model 3** | |
| --- | --- | --- | --- | --- | --- | --- |
|  | **HR (95%CI)^a^** | ***P*** | **HR (95%Cl)^b^** | ***Adjusted P_values_*** | **HR (95%Cl)^c^** | ***Adjusted P_values_*** |
| ***CVD*** |  |  |  |  |  |  |
| **Green tea consumption** |  |  |  |  |  |  |
| Non- consumption | 1 |  | 1 |  | 1 |  |
| Green tea consumers | 0.85 (0.74, 0.97) | 0.019 | 0.77 (0.64, 0.92) | 0.004 | 0.76 (0.63-0.91) | 0.003 |
| **Daily tea leaves consumption** |  |  |  |  |  |  |
| Non- consumption | 1 |  | 1 |  | 1 |  |
| < 2.5 g/day | 0.89 (0.73-1.08) | 0.244 | 0.81 (0.65-1.01) | 0.058 | 0.79 (0.63-0.98) | 0.034 |
| 2.5 – 5 g/day | 0.83 (0.68-1.01) | 0.061 | 0.76 (0.60-0.96) | 0.022 | 0.76 (0.60-0.96) | 0.024 |
| > 5 g/day | 0.84 (0.68-1.03) | 0.098 | 0.73 (0.57-0.94) | 0.013 | 0.71 (0.55-0.92) | 0.008 |
| ***P*** _trend_ |  | 0.051 |  | 0.026 |  | 0.019 |
| **Duration of consumption** |  |  |  |  |  |  |
| Non- consumption | 1 |  | 1 |  | 1 |  |
| < 25 years | 0.63 (0.48-0.81) | < 0.001 | 0.77 (0.59-1.01) | 0.059 | 0.77 (0.58-1.01) | 0.056 |
| 25 – 40 years | 0.83 (0.70-0.999) | 0.049 | 0.84 (0.67-1.06) | 0.141 | 0.82 (0.65-1.03) | 0.089 |
| > 40 years | 1.08 (0.89-1.31) | 0.436 | 0.70 (0.55-0.089) | 0.004 | 0.69 (0.54-0.88) | 0.002 |
| ***P*** _trend_ |  | 0.549 |  | 0.005 |  | 0.003 |
| ***Stroke*** |  |  |  |  |  |  |
| **Green tea consumption** |  |  |  |  |  |  |
| Non- consumption | 1 |  | 1 |  | 1 |  |
| Green tea consumers | 0.85 (0.72-0.998) | 0.047 | 0.78 (0.62-0.97) | 0.024 | 0.77 (0.62-0.96) | 0.02 |
| **Daily tea leaves consumption** |  |  |  |  |  |  |
| Non- consumption | 1 |  | 1 |  | 1 |  |
| < 2.5 g/day | 0.91 (0.71-1.15) | 0.423 | 0.83 (0.64-1.09) | 0.176 | 0.81 (0.62-1.06) | 0.127 |
| 2.5 – 5 g/day | 0.82 (0.64-1.05) | 0.108 | 0.76 (0.57-1.02) | 0.07 | 0.78 (0.58-1.04) | 0.094 |
| > 5 g/day | 0.82 (0.63-1.06) | 0.121 | 0.71 (0.52-0.96) | 0.028 | 0.70 (0.51-0.95) | 0.022 |
| ***P*** _trend_ |  | 0.069 |  | 0.04 |  | 0.036 |
| **Duration of consumption** |  |  |  |  |  |  |
| Non- consumption | 1 |  | 1 |  | 1 |  |
| < 25 years | 0.61 (0.44-0.83) | 0.002 | 0.76 (0.55-1.06) | 0.109 | 0.77 (0.55-1.07) | 0.118 |
| 25 – 40 years | 0.87 (0.70-1.08) | 0.201 | 0.90 (0.69-1.19) | 0.467 | 0.88 (0.67-1.16) | 0.359 |
| > 40 years | 1.03 (0.82-1.31) | 0.781 | 0.67 (0.50-0.90) | 0.008 | 0.67 (0.50-0.90) | 0.008 |
| ***P*** _trend_ |  | 0.556 |  | 0.019 |  | 0.015 |
| ***CHD*** |  |  |  |  |  |  |
| **Green tea consumption** |  |  |  |  |  |  |
| Non- consumption | 1 |  | 1 |  | 1 |  |
| Green tea consumers | 0.77 (0.63-0.95) | 0.013 | 0.70 (0.54-0.93) | 0.013 | 0.68 (0.52-0.90) | 0.006 |
| **Daily tea leaves consumption** |  |  |  |  |  |  |
| Non- consumption | 1 |  | 1 |  | 1 |  |
| < 2.5 g/day | 0.82 (0.60-1.11) | 0.195 | 0.75 (0.53-1.06) | 0.102 | 0.72 (0.51-1.01) | 0.06 |
| 2.5 – 5 g/day | 0.80 (0.59-1.08) | 0.138 | 0.72 (0.50-1.03) | 0.073 | 0.70 (0.49-1.01) | 0.054 |
| > 5 g/day | 0.69 (0.49-0.97) | 0.033 | 0.62 (0.41-0.92) | 0.017 | 0.60 (0.40-0.89) | 0.011 |
| ***P*** _trend_ |  | 0.020 |  | 0.027 |  | 0.020 |
| **Duration of consumption** |  |  |  |  |  |  |
| Non- consumption | 1 |  | 1 |  | 1 |  |
| < 25 years | 0.62 (0.42-0.91) | 0.014 | 0.76 (0.50-1.14) | 0.183 | 0.74 (0.49-1.12) | 0.152 |
| 25 – 40 years | 0.72 (0.54-0.96) | 0.025 | 0.74 (0.52-1.05) | 0.090 | 0.71 (0.49-1.01) | 0.054 |
| > 40 years | 0.98 (0.73-1.33) | 0.901 | 0.64 (0.44-0.92) | 0.016 | 0.61 (0.42-0.88) | 0.009 |
| ***P*** _trend_ |  | 0.158 |  | 0.012 |  | 0.005 |
| Abbreviations: CVD, cardiovascular diseases; CHD, coronary heart disease; HR, hazard ratio; 95%CI: 95% confidence interval. | | | | | | |
| Model 1^a^: Crude model | | | | | | |
| Model 2^b^: Adjusted for covariates in Model 1 as well as age, sex, smoking status, alcohol consumption status, BMI, annual income, education, employment, marital status, physical exercise, SBP, DBP, dyslipidemia, hypertension,lipid-lowering drugs, antihypertensive drugs, oral hypoglycaemic agents, family history of CVD, family history of T2DM, times of weekly meat/fruit/vegetable consumption. | | | | | | |
| Model 3^c^: Adjusted for covariates in Model 2 as well as HbA1c, FPG, diabetes duration, and diabetes complications. | | | | | | |

**Supplementary Table 2:** Sensitivity analysis of the association between daily consumption of green tea leaves and the risk of CVD

|  | **Non- consumption** | **Daily green tea leaves consumption** | | |
| --- | --- | --- | --- | --- |
|  |  | < 2.5 g/day | 2.5 – 5 g/day | > 5 g/day |
| **Adjusted for covariates as well as use insulin versus** | | | | |
| Cases | 579 | 116 | 115 | 105 |
| Person years | 18348.43 | 3959.02 | 4107.92 | 3403.25 |
| Cases/PYs(/1000) | 31.56 | 29.3 | 27.99 | 30.85 |
| HR (95%CI) | Ref | 0.78 (0.63-0.98) | 0.76 (0.59-0.96) | 0.71 (0.55-0.91) |
| **Adjusted for covariates as well as TC and TG** | | | | |
| Cases | 579 | 116 | 115 | 105 |
| Person years | 18348.43 | 3959.02 | 4107.92 | 3403.25 |
| Cases/PYs(/1000) | 31.56 | 29.3 | 27.99 | 30.85 |
| HR (95%CI) | Ref | 0.78 (0.63-0.98) | 0.75 (0.59-0.95) | 0.70 (0.55-0.90) |
| **Replacing the adjustment variable BMI with WC** | | | | |
| Cases | 579 | 116 | 115 | 105 |
| Person years | 18348.43 | 3959.02 | 4107.92 | 3403.25 |
| Cases/PYs(/1000) | 31.56 | 29.3 | 27.99 | 30.85 |
| HR (95%CI) | Ref | 0.79 (0.63-0.99) | 0.76 (0.59-0.96) | 0.71 (0.55-0.92) |
| **Excluding those without center obesity at baseline** | | | | |
| Cases | 364 | 66 | 65 | 66 |
| Person years | 11378.48 | 2315.28 | 2369.98 | 2188.77 |
| Cases/PYs(/1000) | 31.99 | 28.51 | 27.43 | 30.15 |
| HR (95%CI) | Ref | 0.72 (0.54-0.97) | 0.68 (0.50-0.94) | 0.61 (0.44-0.84) |
| Models were adjusted for the same variables in Table 2. | | | | |

**Supplementary Table 3:** Sensitivity analysis of the association between daily consumption of green tea leaves and the risk of stroke

|  | **Non- consumption** | **Daily green tea leaves consumption** | | |
| --- | --- | --- | --- | --- |
|  |  | < 2.5 g/day | 2.5 – 5 g/day | > 5 g/day |
| **Adjusted for covariates as well as use insulin versus** | | | | |
| Cases | 396 | 81 | 78 | 70 |
| Person years | 18348.43 | 3959.02 | 4107.92 | 3403.25 |
| Cases/PYs(/1000) | 21.58 | 20.46 | 18.99 | 20.57 |
| HR (95%CI) | Ref | 0.81 (0.62-1.06) | 0.78 (0.58-1.04) | 0.69 (0.51-0.94) |
| **Adjusted for covariates as well as TC and TG** | | | | |
| Cases | 396 | 81 | 78 | 70 |
| Person years | 18348.43 | 3959.02 | 4107.92 | 3403.25 |
| Cases/PYs(/1000) | 21.58 | 20.46 | 18.99 | 20.57 |
| HR (95%CI) | Ref | 0.81 (0.62-1.06) | 0.76 (0.57-1.02) | 0.68 (0.50-0.93) |
| **Replacing the adjustment variable BMI with WC** | | | | |
| Cases | 396 | 81 | 78 | 70 |
| Person years | 18348.43 | 3959.02 | 4107.92 | 3403.25 |
| Cases/PYs(/1000) | 21.58 | 20.46 | 18.99 | 20.57 |
| HR (95%CI) | Ref | 0.82 (0.62-1.07) | 0.77 (0.57-1.03) | 0.69 (0.51-0.94) |
| **Excluding those without center obesity at baseline** | | | | |
| Cases | 252 | 43 | 42 | 48 |
| Person years | 11378.48 | 2315.28 | 2369.98 | 2188.77 |
| Cases/PYs(/1000) | 22.15 | 18.57 | 17.72 | 21.93 |
| HR (95%CI) | Ref | 0.67 (0.46-0.96) | 0.63 (0.43-0.93) | 0.61 (0.41-0.90) |
| Models were adjusted for the same variables in Table 2. | | | | |

**Supplementary Table 4**: Sensitivity analysis of the association between daily consumption of green tea leaves and the risk of CHD

|  | **Non- consumption** | **Daily green tea leaves consumption** | | |
| --- | --- | --- | --- | --- |
|  |  | < 2.5 g/day | 2.5 – 5 g/day | > 5 g/day |
| **Adjusted for covariates as well as use insulin versus** | | | | |
| Cases | 261 | 48 | 50 | 39 |
| Person years | 18348.43 | 3959.02 | 4107.92 | 3403.25 |
| Cases/PYs(/1000) | 14.22 | 12.12 | 12.17 | 11.46 |
| HR (95%CI) | Ref | 0.72 (0.51-1.01) | 0.70 (0.49-1.01) | 0.59 (0.40-0.89) |
| **Adjusted for covariates as well as TC and TG** | | | | |
| Cases | 261 | 48 | 50 | 39 |
| Person years | 18348.43 | 3959.02 | 4107.92 | 3403.25 |
| Cases/PYs(/1000) | 14.22 | 12.12 | 12.17 | 11.46 |
| HR (95%CI) | Ref | 0.72 (0.51-1.01) | 0.69 (0.48-0.99) | 0.59 (0.40-0.88) |
| **Replacing the adjustment variable BMI with WC** | | | | |
| Cases | 261 | 48 | 50 | 39 |
| Person years | 18348.43 | 3959.02 | 4107.92 | 3403.25 |
| Cases/PYs(/1000) | 14.22 | 12.12 | 12.17 | 11.46 |
| HR (95%CI) | Ref | 0.73 (0.52-1.03) | 0.72 (0.50-1.03) | 0.62 (0.42-0.92) |
| **Excluding those without center obesity at baseline** | | | | |
| Cases | 162 | 31 | 31 | 22 |
| Person years | 11378.48 | 2315.28 | 2369.98 | 2188.77 |
| Cases/PYs(/1000) | 14.24 | 13.39 | 13.08 | 10.05 |
| HR (95%CI) | Ref | 0.77 (0.50-1.19) | 0.69 (0.43-1.10) | 0.47 (0.27-0.80) |
| Models were adjusted for the same variables in Table 2. | | | | |

**Supplementary Table 5:** Univariate association between traditional risk factors and the risk of CVD, stroke, and CHD

|  | **CVD** | | **Stroke** | | **CHD** | |
| --- | --- | --- | --- | --- | --- | --- |
|  | **HR (95%CI)** | ***P*** | **HR (95%CI)** | ***P*** | **HR (95%CI)** | ***P*** |
| Sex | 0.98 (0.86, 1.12) | 0.763 | 0.97 (0.82, 1.10) | 0.686 | 0.89 (0.73, 1.10) | 0.27 |
| Age at baseline | 1.05 (1.04, 1.06) | < 0.001 | 1.06 (1.00, 1.10) | < 0.001 | 1.05 (1.00, 1.10) | < 0.001 |
| BMI | 1.02 (1.00, 1.1) | 0.09 | 1.02 (0.99, 1.10) | 0.152 | 1.05 (1.00, 1.10) | 0.015 |
| Smoking | 1.01 (0.86, 1.18) | 0.906 | 0.95 (0.78, 1.20) | 0.601 | 0.999 (0.79, 1.30) | 0.994 |
| Drinking | 0.87 (0.74, 1.03) | 0.098 | 0.89 (0.73, 1.10) | 0.233 | 0.78 (0.61, 1.00) | 0.059 |
| Physical exercise | 1.00 (0.86, 1.17) | 0.981 | 0.87 (0.72, 1.10) | 0.177 | 1.24 (0.99, 1.50) | 0.064 |
| Red meats consumption | 0.68 (0.59, 0.789) | < 0.001 | 0.69 (0.58, 0.82) | < 0.001 | 0.59 (0.47, 0.74) | < 0.001 |
| Fruits consumption | 0.72 (0.60, 0.861) | < 0.001 | 0.65 (0.52, 0.81) | 0 | 0.87 (0.68, 1.10) | 0.293 |
| Vegetable consumption | 1.01 (0.77, 1.31) | 0.968 | 0.95 (0.69, 1.30) | 0.758 | 1.13 (0.74, 1.70) | 0.567 |
| Family history of CVD | 0.82 (0.62, 1.08) | 0.156 | 0.68 (0.47, 0.98) | 0.041 | 0.91 (0.61, 1.40) | 0.652 |
| Family history of T2DM | 0.82 (0.62, 1.08) | 0.156 | 0.68 (0.47, 0.98) | 0.041 | 0.91 (0.61, 1.40) | 0.652 |
| Dyslipidemia | 1.03 (0.91, 1.18) | 0.611 | 1.09 (0.93, 1.30) | 0.293 | 0.96 (0.79, 1.20) | 0.654 |
| Hypertension | 2.16 (1.69, 2.76) | < 0.001 | 2.64 (1.90, 3.70) | < 0.001 | 1.88 (1.30, 2.70) | < 0.001 |
| Oral hypoglycaemic agents | 1.71 (1.42, 2.04) | < 0.001 | 1.97 (1.56, 2.48) | < 0.001 | 1.38 (1.07, 1.78) | 0.014 |
| Antihypertensive drugs | 1.91 (1.64, 2.23) | < 0.001 | 1.94 (1.61, 2.34) | < 0.001 | 1.97 (1.56, 2.50) | < 0.001 |
| Lipid-lowering drugs | 1.50 (1.06, 2.14) | 0.024 | 1.59 (1.05, 2.41) | 0.030 | 1.28 (0.72, 2.27) | 0.405 |
| diabetes duration | 1.03 (1.02, 1.04) | < 0.001 | 1.03 (1.00, 1.00) | < 0.001 | 1.04 (10, 1.10) | < 0.001 |
| Diabetes complications | 1.71 (1.42, 2.04) | < 0.001 | 1.97 (1.56, 2.48) | < 0.001 | 1.38 (1.07, 1.78) | 0.014 |
| Fasting plasma glucose | 1.02 (0.996, 1.05) | 0.108 | 1.03 (1.00, 1.06) | 0.038 | 1.00 (0.97, 1.04) | 0.854 |
| HbA1c | 1.11 (1.06, 1.15) | < 0.001 | 1.15 (1.10, 1.20) | < 0.001 | 1.06 (0.99, 1.10) | 0.08 |
| Abbreviations: CVD, cardiovascular diseases; CHD, coronary heart disease; HR, hazard ratio;95%CI, 95% confidence interval. | | | | | | |

**Supplementary Table 6:** Sensitivity analyses of E-value

|  | **CVD** | **Stroke** | **CHD** |
| --- | --- | --- | --- |
|  | **E -value (95%CI)** | **E -value (95%CI)** | **E -value (95%CI)** |
| Tea consumption |  |  |  |
| Non-drinker | - | - | - |
| Non-fermented tea | 1.96 (1.43) | 1.92 (1.25) | 2.30 (1.46) |
| Daily consumption (grams/day) |  |  |  |
| Non-drinker | - | - | - |
| < 2.5 | 1.85 (1.16) | - | - |
| 2.5 - 5 | 1.96 (1.25) | - | - |
| > 5 | 2.17 (1.39) | 2.21 (1.29) | 2.72 (1.50) |
| ***P*** _trend_ |  |  |  |
| Duration of consumption (years) |  |  |  |
| Non-drinker | - | - | - |
| <25 | - | - | - |
| 25 - 40 | - | - | - |
| >40 | 2.26 (1.53) | 2.35 (1.46) | 2.66 (1.53) |
| ***P*** _trend_ |  |  |  |
| Abbreviations: CVD, cardiovascular diseases; CHD, coronary heart disease. | | | |
| Adjusted for covariates in age, sex, smoking status, alcohol consumption status, BMI, annual income, education, employment, marital status, physical exercise, SBP, DBP, dyslipidemia, hypertension,lipid-lowering drugs, antihypertensive drugs, oral hypoglycaemic agents, family history of CVD, family history of T2DM, times of weekly meat/fruit/vegetable consumption, HbA1c, FPG, diabetes duration, and diabetes complications. | | | |

**Supplementary Table 7:** Sensitivity analyses of the association between the duration of green tea consumption and the risk of CVD

|  | **Non- consumption** | **Duration of consumption** | | |
| --- | --- | --- | --- | --- |
|  |  | < 25 years | 25 - 40 years | > 40 years |
| **Adjusted for covariates as well as use insulin versus** | | | | |
| Cases | 579 | 65 | 146 | 125 |
| Person years | 18348.43 | 2976.5 | 5203.92 | 3289.77 |
| Cases/PYs(/1000) | 31.56 | 21.84 | 28.06 | 38 |
| HR (95%CI) | Ref | 0.76 (0.58-1.00) | 0.82 (0.65-1.03) | 0.69 (0.54-0.88) |
| **Adjusted for covariates as well as TC and TG** | | | | |
| Cases | 579 | 65 | 146 | 125 |
| Person years | 18348.43 | 2976.5 | 5203.92 | 3289.77 |
| Cases/PYs(/1000) | 31.56 | 21.84 | 28.06 | 38 |
| HR (95%CI) | Ref | 0.76 (0.58-1.00) | 0.82 (0.65-1.02) | 0.68 (0.53-0.86) |
| **Replacing the adjustment variable BMI with WC** | | | | |
| Cases | 579 | 65 | 146 | 125 |
| Person years | 18348.43 | 2976.5 | 5203.92 | 3289.77 |
| Cases/PYs(/1000) | 31.56 | 21.84 | 28.06 | 38 |
| HR (95%CI) | Ref | 0.77 (0.59-1.01) | 0.82 (0.66-1.04) | 0.69 (0.54-0.87) |
| **Excluding those without center obesity at baseline** | | | | |
| Cases | 364 | 38 | 82 | 77 |
| Person years | 11378.48 | 1873.74 | 2988.28 | 2012.01 |
| Cases/PYs(/1000) | 31.99 | 20.28 | 27.44 | 38.27 |
| HR (95%CI) | Ref | 0.68 (0.48-0.97) | 0.73 (0.54-1.00) | 0.62 (0.45-0.86) |
| Models were adjusted for the same variables in Table 2. | | | | |

**Supplementary Table 8:** Sensitivity analyses of the association between duration of green tea consumption and the risk of stroke

|  | **Non- consumption** | **Duration of consumption** | | |
| --- | --- | --- | --- | --- |
|  |  | < 25 years | 25 - 40 years | > 40 years |
| **Adjusted for covariates as well as use insulin versus** | | | | |
| Cases | 396 | 43 | 104 | 82 |
| Person years | 18348.43 | 2976.5 | 5203.92 | 3289.77 |
| Cases/PYs(/1000) | 21.58 | 14.45 | 19.98 | 24.93 |
| HR (95%CI) | Ref | 0.76 (0.55-1.06) | 0.88 (0.67-1.15) | 0.67 (0.50-0.90) |
| **Adjusted for covariates as well as TC and TG** | | | | |
| Cases | 396 | 43 | 104 | 82 |
| Person years | 18348.43 | 2976.5 | 5203.92 | 3289.77 |
| Cases/PYs(/1000) | 21.58 | 14.45 | 19.98 | 24.93 |
| HR (95%CI) | Ref | 0.76 (0.55-1.06) | 0.87 (0.66-1.15) | 0.66 (0.49-0.88) |
| **Replacing the adjustment variable BMI with WC** | | | | |
| Cases | 396 | 43 | 104 | 82 |
| Person years | 18348.43 | 2976.5 | 5203.92 | 3289.77 |
| Cases/PYs(/1000) | 21.58 | 14.45 | 19.98 | 24.93 |
| HR (95%CI) | Ref | 0.77 (0.55-1.07) | 0.88 (0.67-1.15) | 0.66 (0.49-0.89) |
| **Excluding those without center obesity at baseline** | | | | |
| Cases | 252 | 23 | 61 | 49 |
| Person years | 11378.48 | 1873.74 | 2988.28 | 2012.01 |
| Cases/PYs(/1000) | 22.15 | 12.27 | 20.41 | 24.35 |
| HR (95%CI) | Ref | 0.59 (0.37-0.92) | 0.77 (0.53-1.11) | 0.57 (0.38-0.83) |
| Models were adjusted for the same variables in Table 2. | | | | |

**Supplementary Table 9:** Sensitivity analyses of the association between duration of green tea consumption and the risk of CHD

|  | **Non- consumption** | **Duration of consumption** | | |
| --- | --- | --- | --- | --- |
|  |  | < 25 years | 25 - 40 years | > 40 years |
| **Adjusted for covariates as well as use insulin versus** | | | | |
| Cases | 261 | 29 | 57 | 51 |
| Person years | 18348.43 | 2976.5 | 5203.92 | 3289.77 |
| Cases/PYs(/1000) | 14.22 | 9.74 | 10.95 | 15.5 |
| HR (95%CI) | Ref | 0.74 (0.49-1.12) | 0.71 (0.49-1.01) | 0.61 (0.42-0.88) |
| **Adjusted for covariates as well as TC and TG** | | | | |
| Cases | 261 | 29 | 57 | 51 |
| Person years | 18348.43 | 2976.5 | 5203.92 | 3289.77 |
| Cases/PYs(/1000) | 14.22 | 9.74 | 10.95 | 15.5 |
| HR (95%CI) | Ref | 0.74 (0.49-1.11) | 0.70 (0.49-1.00) | 0.60 (0.41-0.87) |
| **Replacing the adjustment variable BMI with WC** | | | | |
| Cases | 261 | 29 | 57 | 51 |
| Person years | 18348.43 | 2976.5 | 5203.92 | 3289.77 |
| Cases/PYs(/1000) | 14.22 | 9.74 | 10.95 | 15.5 |
| HR (95%CI) | Ref | 0.76 (0.51-1.14) | 0.73 (0.51-1.04) | 0.62 (0.43-0.90) |
| **Excluding those without center obesity at baseline** | | | | |
| Cases | 162 | 18 | 34 | 32 |
| Person years | 11378.48 | 1873.74 | 2988.28 | 2012.01 |
| Cases/PYs(/1000) | 14.24 | 9.61 | 11.38 | 15.9 |
| HR (95%CI) | Ref | 0.72 (0.43-1.20) | 0.70 (0.44-1.13) | 0.58 (0.36-0.94) |
| Models were adjusted for the same variables in Table 2. | | | | |

**Supplementary Table 10:** Association between other types of tea consumption and the risk of CVD stroke and CHD

|  | **Cases** | **Cases/ PYs (/1000)** | **HR (95%Cl)** | ***Adjusted P_values_*** |
| --- | --- | --- | --- | --- |
| Total CVD |  |  |  |  |
| Type of tea consumption |  |  |  |  |
| Non- consumption | 579 | 31.56 |  |  |
| Both intakes | 37 | 31.25 | 1.24 (0.86-1.78) | 0.253 |
| Fermented tea | 15 | 40.46 | 1.10 (0.65-1.84) | 0.732 |
| Stroke |  |  |  |  |
| Type of tea consumption |  |  |  |  |
| Non- consumption | 396 | 21.58 | 1 |  |
| Both intakes | 24 | 20.27 | 1.15 (0.73-1.80) | 0.544 |
| Fermented tea | 10 | 26.97 | 1.08 (0.57-2.05) | 0.806 |
| CHD |  |  |  |  |
| Type of tea consumption |  |  |  |  |
| Non- consumption | 261 | 14.22 | 1 |  |
| Both intakes | 13 | 10.98 | 1.03 (0.57-1.88) | 0.920 |
| Fermented tea | 7 | 18.88 | 1.10 (0.51-2.37) | 0.802 |
